# Assessing the severity of positive valence symptoms in initial psychiatric evaluation records: Should we use convolutional neural networks?

**DOI:** 10.1371/journal.pone.0204493

**Published:** 2018-10-16

**Authors:** Hong-Jie Dai, Jitendra Jonnagaddala

**Affiliations:** 1 Department of Computer Science and Information Engineering, National Taitung University, Taitung, Taiwan; 2 Interdisciplinary Program of Green and Information Technology, National Taitung University, Taitung, Taiwan; 3 School of Public Health and Community Medicine, UNSW Sydney, Australia; Boston Children's Hospital / Harvard Medical School, UNITED STATES

## Abstract

**Background and objective:**

Efficiently capturing the severity of positive valence symptoms could aid in risk stratification for adverse outcomes among patients with psychiatric disorders and identify optimal treatment strategies for patient subgroups. Motivated by the success of convolutional neural networks (CNNs) in classification tasks, we studied the application of various CNN architectures and their performance in predicting the severity of positive valence symptoms in patients with psychiatric disorders based on initial psychiatric evaluation records.

**Methods:**

Psychiatric evaluation records contain unstructured text and semi-structured data such as question–answer pairs. For a given record, we tokenise and normalise the semi-structured content. Pre-processed tokenised words are represented as one-hot encoded word vectors. We then apply different configurations of convolutional and max pooling layers to automatically learn important features from various word representations. We conducted a series of experiments to explore the effect of different CNN architectures on the classification of psychiatric records.

**Results:**

Our best CNN model achieved a mean absolute error (MAE) of 0.539 and a normalized MAE of 0.785 on the test dataset, which is comparable to the other well-known text classification algorithms studied in this work. Our results also suggest that the normalisation step has a great impact on the performance of the developed models.

**Conclusions:**

We demonstrate that normalisation of the semi-structured contents can improve the MAE among all CNN configurations. Without advanced feature engineering, CNN-based approaches can provide a comparable solution for classifying positive valence symptom severity in initial psychiatric evaluation records. Although word embedding is well known for its ability to capture relatively low-dimensional similarity between words, our experimental results show that pre-trained embeddings do not improve the classification performance. This phenomenon may be due to the inability of word embeddings to capture problem specific contextual semantic information implying the quality of the employing embedding is critical for obtaining an accurate CNN model.

## 1 Introduction

Approximately 18% of adults have been identified as meeting the criteria of at least one common mental disorder [1]. In addition, psychiatric disorders can lead to a two-fold higher mortality rate compared to the mentally healthy population and contribute to around 14.3% of deaths worldwide. The median potential life lost due to psychiatric disorders has been estimated to be 10 years [2].

Although pharmacological treatments for mental illness have developed rapidly in recent decades, the response rate is still unsatisfactory and unpredictable. One major concern is that diagnoses of psychiatric disorders are based on behavioural symptoms and signs, which may be insufficiently precise and objective. To improve the precision of psychiatric diagnoses, the National Institute of Mental Health (NIMH) initiated the Research Domain Criteria (RDoC) project to develop a new approach to classifying psychiatric disorders according to diverse neurobiological measures—including biomarkers, genetics, and neuroimaging—in addition to clinical symptoms [3, 4]. The RDoC framework introduces an RDoC matrix consisting of the following five psychiatric research domains. The objective of this framework is to facilitate better understanding of the basic dimensions of functionality that underlie changes in human behaviours.

- Positive valence systems are primarily responsible for responses to positive motivational situations or contexts, such as reward seeking.
- Negative valence systems are primarily responsible for responses to aversive situations or context, such as anxiety and loss.
- Cognitive systems are responsible for various cognitive processes.
- Social processing systems mediate responses to interpersonal settings of various types, including the perception and interpretation of others’ actions.
- Arousal/regulatory systems are responsible for generating appropriate activation and homeostatic regulation of neural systems.

Each of these domains is characterised using available data (i.e. genomic, molecular, cellular, circuital, physiological, behavioural, self-reported, and paradigmatic) to examine patients’ health and psychiatric illnesses from cross-diagnostic perspectives. For example, initial psychiatric records are more likely to contain references of behaviours or self-reports compared to those of genes or molecules.

The positive valence domain pertains to events, objects, or situations which are attractive to the patients to the point at which they are willing to be actively engaged. Psychiatric disorders resulting from abnormalities of positive valence systems include mania, substance abuse and dependence, obsessive-compulsive disorder, and depression. The ability to efficiently capture this domain should facilitate efforts to stratify the risk of adverse outcomes among patients with psychiatric disorders, as well as to identify optimal treatment strategies for patient subgroups [5].

Severity classification is essential, as it may help in determining whether a patient requires special medical attention or hospitalisation. In this work, we develop classification models for categorizing the severity of positive valence symptoms based on a dataset of initial psychiatric evaluation records. We employ deep convolutional neural networks (CNN), which have achieved great success in image classification challenges [6] and are at the core of most current computer vision systems. Several recent studies have applied CNNs to problems in natural language processing [7–10] with intriguing results. Specifically, Tran and Kavuluru [11] demonstrated that CNN models can predict a few mental conditions based on the short history of present illness segments in psychiatric notes. Gkotsis, Oellrich [12] and Orabi, Buddhitha [13] developed methods to analyze and classify posts from social media platforms that are related to mental illness and depression respectively, and observed that CNN models achieved the best performance. The success in the text classification task suggests that CNNs may serve as drop-in replacements for baseline models. Therefore, we decide to investigate the potential of adapting the conventional CNN architectures to the problem of severity classification of psychiatric notes. Unlike previous severity classification works which tried to optimize the classification performance by conducting feature engineering [14, 15] or ensemble learning [14, 16, 17], we studied the performance of CNN-based text classification architecture and its variants to the severity classification problem in comparison to that of well-established baseline models such as C4.5, Support Vector Machine, and Naïve Bayes Multinomial. In this study we also aimed to investigate the ability of CNN-based models in ingesting the free text of psychiatric records without pre-processing them by extracting semi-structured/template parts as additional features in our CNN-based models.

## 2 Methods

### 2.1 Dataset

The Center of Excellence in Genomic Science (CEGS) Neuropsychiatric Genome-scale and RDoC Individualized Domains (N-GRID) dataset was used in this study [18]. The dataset contains de-identified initial psychiatric evaluation records collected from Partners Healthcare and the N-GRID project of Harvard Medical School on a per-patient basis [5]. A total of 649 records were annotated manually with the following classes by psychiatric experts with several years of clinical experience:

- ABSENT: no symptoms presented
- MILD: some symptoms presented without a focus of treatment
- MODERATE: symptoms presented with a focus of treatment but do not require hospitalisation
- SEVERE: symptoms presented requiring hospitalisation or emergency room visit, or will otherwise result in major clinical consequences

The annotated data were split into training and test sets containing 433 and 216 records, respectively. Table 1 summarises the details of the dataset. This dataset has been used to predict the mental health condition of a patient [19]. In an another study, this dataset is used to analyse associations between clinical/social parameters and violent behaviour in patients with psychiatric disorders [20]. Please refer to S1 and S2 Figs and the work represented by Uzuner, Stubbs [5] for the demographic information and details on the manual annotations of the dataset.

**Table 1:** Record distribution of the CEGS N-GRID 2016 dataset.

Type	Absent	Mild	Moderate	Severe
Training Set	61 (14.08%)	166 (38.33%)	110 (25.4%)	96 (22.17%)
Test Set	31 (14.35%)	86 (39.81%)	46 (21.3%)	53 (24.54%)

Records of psychiatric evaluations in the released dataset contained a variety of text formats. For example, most of the records contain a set of pre-defined questions which are described as question–answer pairs, whose answers may either be short (e.g., ‘Yes’ or ‘No’), or more verbose natural language descriptions from the patient as written by psychiatrists. Such descriptions may contain further comment from psychiatrists that may be crucial to the evaluation of the patient’s mental state. In summary, three formats can be observed in a single record: 1) Narrative text that includes section headings and unstructured text; 2) semi-structured text, such as attribute–value pairs, which often occur in lists; and 3) text templates that consist of a heading followed by a variety of question–answer pairs to assess the patient.

### 2.2 Pre-processing

The dataset was pre-processed to generate tokens and reduce the effect of noise, including misspellings. First, Hunspell downloaded from http://hunspell.github.io/ was used to correct frequently misspelled words found in a given record. The clinical natural language processing library developed in our previous work [21] was then used to split the text into sentences and tokens. After manually checking the pre-processed results, we observed that the dataset contains words which were merged together. For instance, the words ‘PsychiatryChief’ and ‘..JMH.PatientDose..’ should be ‘Psychiatry Chief’ and ‘..JMH. Patient dose..’, respectively. Therefore, we developed rules that were executed after the tokenisation step to refine the tokenisation results. For example, a token matching patterns like ‘[a-z][A-Z]’ and ‘\d[A-z]’ is separated into two tokens. Finally, semi-structured text and templates such as attribute–value and question–answer pairs were extracted using regular expression patterns. For each pair, the question was normalised into a short form in order of appearance.

### 2.3 Convolutional neural network model

Although the corpus used was annotated with a single value indicating the severity of positive valence symptoms, the supporting textual evidence upon which these judgements were made was not annotated. Therefore, we formulated the problem as a document classification problem to which we adapted a CNN.

**Fig 1:**
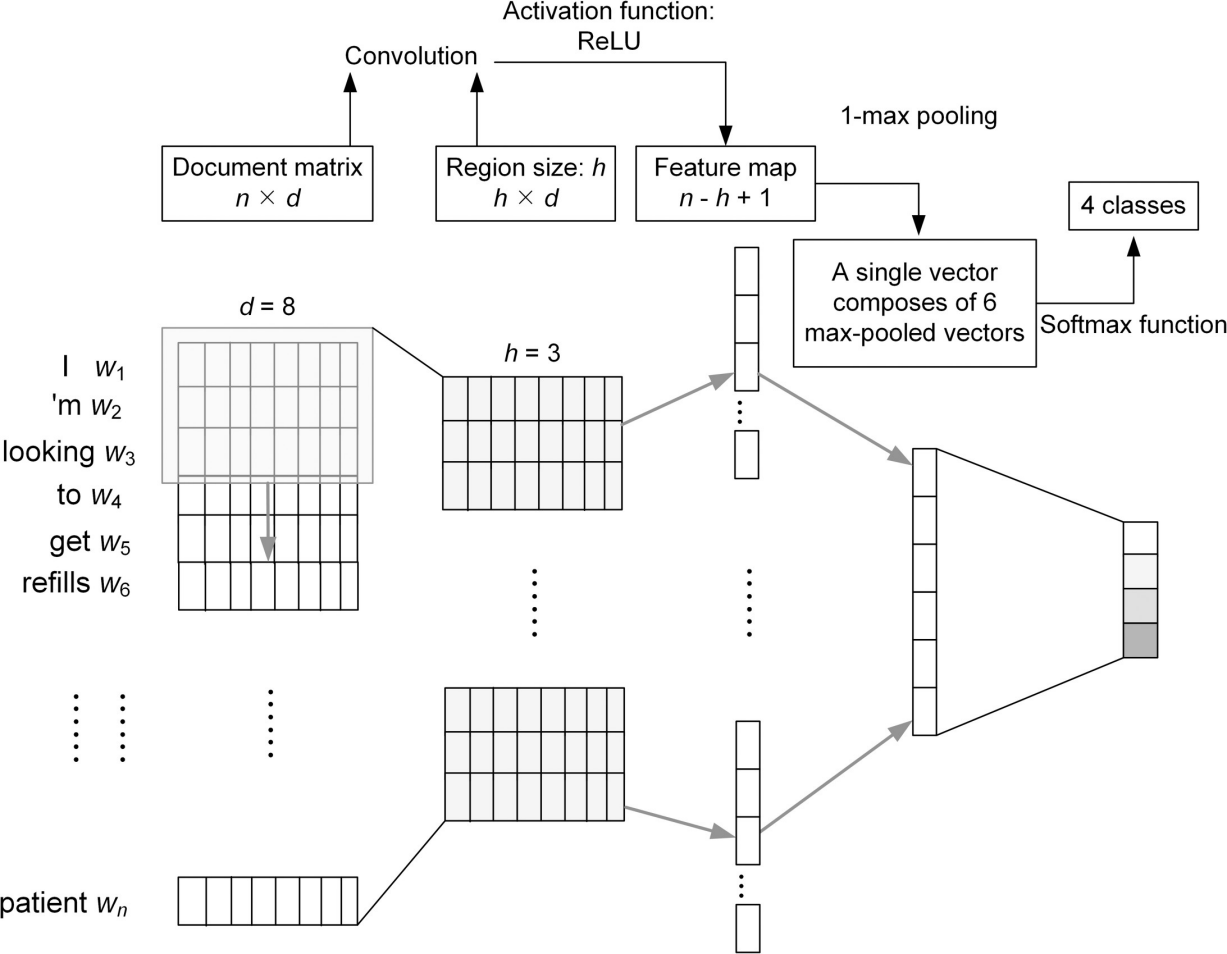
A basic deep convolutional neural network based severity classification model.

A CNN is a feed-forward neural network with convolution layers interleaved with pooling layers. Fig 1 shows a typical CNN used for document classification [7, 8, 22]. In image classification tasks, the CNN input are image pixel data; analogous to pixels in an image, each word in a psychiatric evaluation record can be considered as a pixel. In our implementation, the input words were supplied in the same order as observed in the original psychiatric records because we believe that preserving the order is important for neural networks to capture the semantic meaning embedded in the record. All existing words in a record are then represented as a word matrix, in which each row is a vector corresponding to one word appearing in the record in one-hot form, and the dimension of the vector is equal to the vocabulary size. These vectors are mapped to low-dimensional representations through a matrix product. Let *w*_*i*_ ∈ R^*d*^ be the *d*-dimensional word embedding vector corresponding to the *i*th word of the input sequence. The input text **w** of length *n* can be represented as Eq 1.

$$w_{1:n}=w_{1}⨁w_{2}⨁\ldots ⨁w_{n}$$ (1)

The embedding layer then goes through the convolution layer, which applies convolutions over the embedded input to compute its output. In image processing, convolution refers to the process of adding the value of each element of an image to its local neighbouring elements’ values weighted by a kernel that is also known as a filter or convolution matrix. Depending on the kernel, convolutions can cause a wide range of effects in image processing, such as blurring, sharpening, or edge detection. To employ this concept in our document classification problem, a convolution can be considered to execute a sliding window function over full rows of the word matrix. This sliding window serves a similar function of the kernel during image processing, working as a feature extractor that can capture implicit linguistic properties buried in the word matrix.

In Fig 1, the filter height of a filter (denoted as *h*), commonly called the region size of the filter [7], is set to three, and the width is equal to that of the word matrix. The filter function as defined in Eq 2 multiplies its values element-wise with the original word matrix and sums them, and then slides over the whole matrix to obtain the convolution result.

$$c_{i}=f(w∙x_{i:i+h-1}+b)$$ (2)

Eq 2 shows the feature *c*_*i*_ generated from a window of words *x*_*i*:*i+h*-1_. Here *b* is a bias value and *f* is a non-linear function. This function is applied to each possible window in the input sequence {*x*_1:*h*_, *x*_2:*h*+1_, …, *x*_*n*-*h*+1:*n*_} by multiplying its values element-wise with the words in the window and sums them up. It then slides over the whole matrix to obtain the convolution result, which is referred to as a feature map **c** as defined in Eq 3.

$$c=[c_{1},c_{2},\ldots ,c_{n-h+1}]$$ (3)

The convolution step indicated in Fig 1 connects each region, composed of three-word vectors of the input matrix, to one output neuron. As shown in Fig 1, we applied the most popular activation function for deep neural networks, the rectified linear unit (ReLU) [23], to each output of the convolutional layer.

The max-pooling layer following the convolutional layer subsamples the feature map (the output from the convolution step) to output the maximum activation value by Eq 4.

$$\hat{c}=\max(c)$$ (4)

For each feature map, the layer outputs the maximum activation values based on window size to produce a fixed-length vector composed of the most important features. The layer can also mitigate overfitting during training by determining the dimensions of the outputs from filters and selecting the most prominent information from the convolutional layer. Fig 1 demonstrates a 1-max pooling strategy commonly used in sentence classification to generate the largest number from six feature maps.

Finally, similar to regular neural networks, all outputs from the max-pooling layer are concatenated to form a fixed-length feature vector, which is then fully connected to a softmax layer output a probability distribution for the four possible classes.

### 2.4 Variations of CNN models

The basic CNN document model displayed in Fig 1 can have a variety of different architectures. First, the word embedding layer can either be initialised with word vectors obtained from an unsupervised neural language model trained by a given unlabelled dataset, or it can be randomly initialised and then modified during training. One can also combine both word vectors into two channels, with one kept static throughout training and the other fine-tuned via backpropagation.

**Fig 2:**
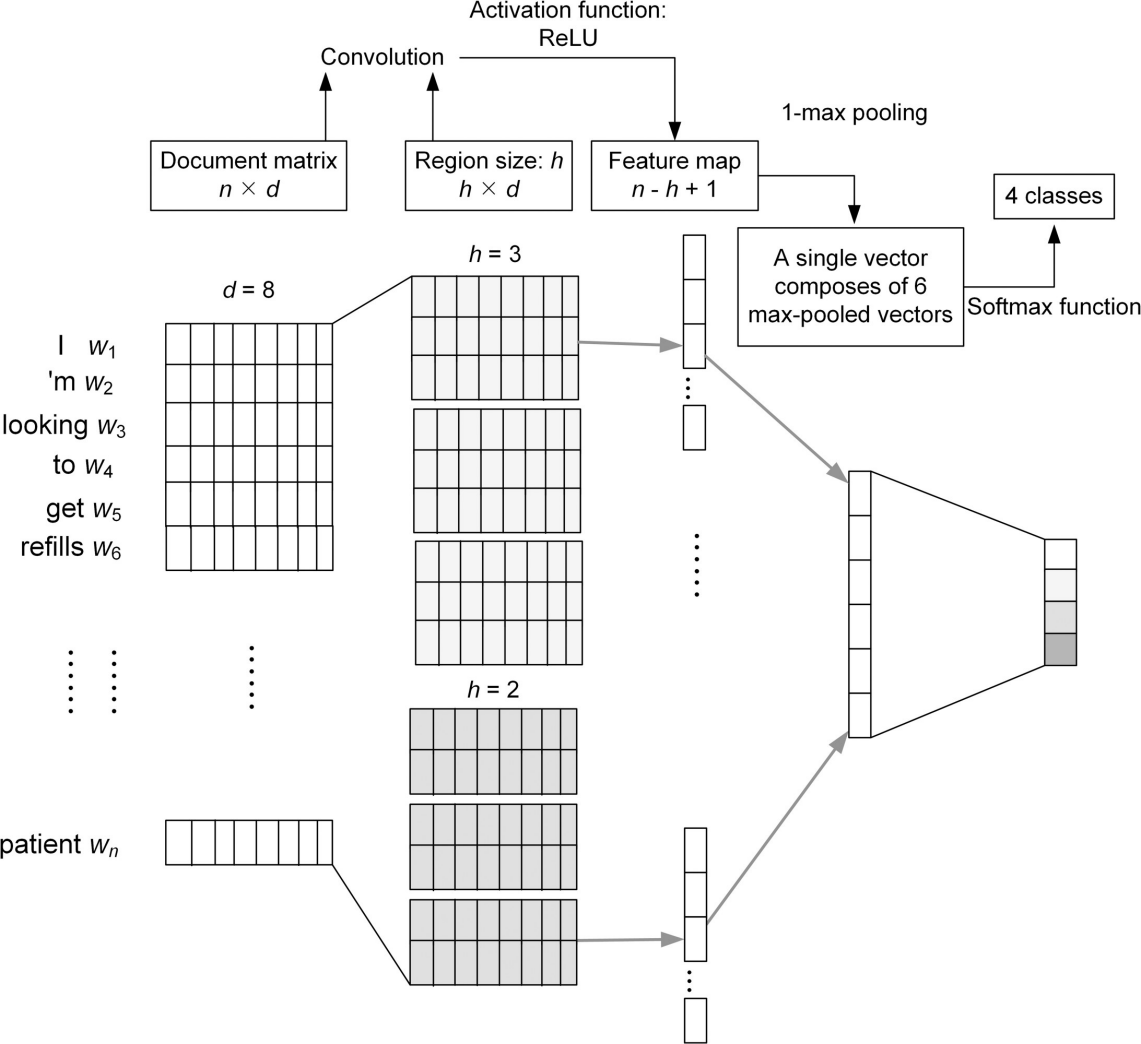
A parallel CNN model with two filters for two region sizes (*h* = 2 and 3) and 1-max pooling.

In Fig 1, we showed multiple filters for the same region size (*h* = 3) to learn complementary features from the same regions. By contrast, a parallel CNN [10] utilises multiple filters with different region sizes, as illustrated in Fig 2. The outputs of all max-pooling layers are stacked together as the input to the final layer.

**Fig 3:**
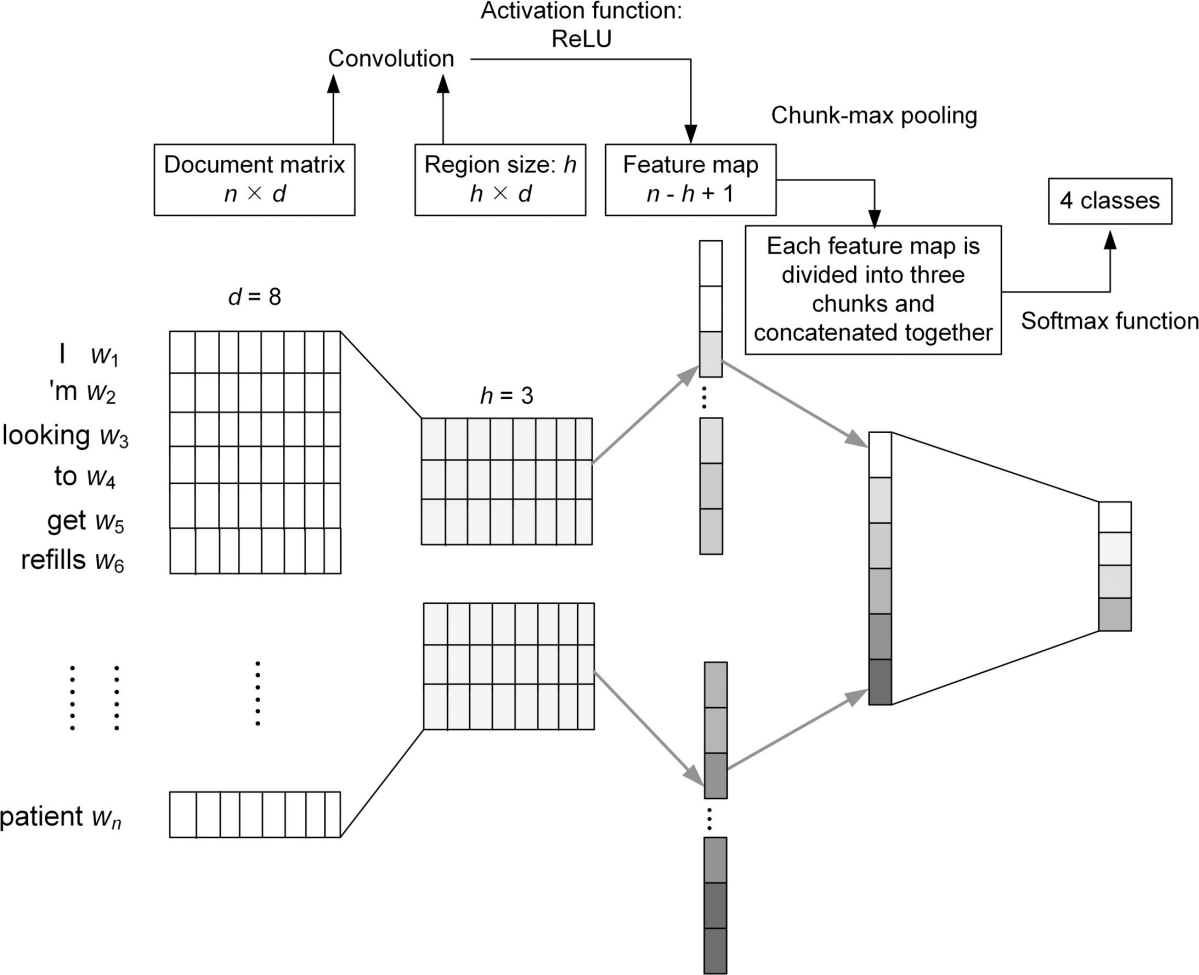
A CNN model with chunk-max pooling.

The model can be further extended by segmenting the feature maps into several chunks, and then performing max pooling over each chunk to generate the top *n* features. As illustrated in Fig 3, the first feature map outputs **c**_**1**_ is divided into three chunks *c*_11_, *c*_12_, *c*_13_. Output of the chunk-pooling can be expressed as Eq 5, where *i* indicates the max-pool results of the *i*th feature map and *l* is the number of chunk.

$$\hat{c_{i}}=\max(c_{i1})⨁\max(c_{i2})⨁\cdots ⨁\max(c_{il})$$ (5)

Through the chunk-max pooling layer, we obtain the $\hat{c_{i}}$ for each feature map. Then, we concatenate all max-pooled chunks to form the vector for fully connected layer. We refer to this architecture as a chunk-max pooling CNN [24].

### 2.5 Evaluation and performance measures

We assessed the performance of several variations of CNN models using a stratified five-fold cross-validation (CV) on the training set. Additionally, the performance of CNN models was assessed independently using the test set. The mean absolute error (MAE) metric, defined as Eq 6, was used to evaluate the model performance for each class. MAE was chosen instead of the other metrics because we would like to quantify the distance between the predicted and the gold standard classes. We further used the macro-averaged MAE (Eq 7) to report the overall cross-class performance to avoid a class-imbalance bias as shown in Table 1.

$$MAE(D_{j})=\frac{1}{\left|D_{j}\right|}\sum_{x_{i}\in D_{j}}\left|p(x_{i})-y_{i}\right|$$ (6)

$$MMAE(C,D)=\frac{1}{\left|C\right|}\sum_{j=1}^{\left|C\right|}MAE(D_{j})$$ (7)

where *C* is the set of classes {ABSENT, MILD, MODERATE, SEVERE}. The score corresponding to each class is the same as its index (i.e. the scores for ‘absent’ and ‘severe’ are 0 and 3, respectively). *D*_*j*_ is the set of records with the *j*^th^ class, and *x*_*i*_ is a record from *D*_*j*_. *p*(*x*_*i*_) represents the predicted score of *x*_*i*_, which can be inferred by checking the index of the predicted class in *C*. *y*_*i*_ indicates the score of the manual annotation done by annotators corresponding to *x*_*i*_.

## 3 Results

### 3.1 Baseline performance and the effect of pre-processing

We first evaluated the performance of well-known text classification algorithms with bag-of-word features on the training set with stratified five-fold CV. C4.5 [25], support vector machine (SVM) [26], and naïve Bayes multinomial (NBM) [27] algorithms were used in this experiment. For each baseline algorithm, we measured the information gain of each feature with respect to the four classes by Eq. A.1 and filtered out lower-ranking features. The detail of the employed feature selection algorithm can be found in the Appendix A in S1 File. The basic CNN model used for comparison was configured with 100 filters with a region size of 1. The learning rate and epoch were set to 0.001 and 150, respectively. The dimension of the word embedding layer of the CNN model was set to 200 with values initialised from a uniform distribution.

The same pre-processing steps described in the previous section were applied for all algorithms to generate two pre-processed datasets: one with only typo correction and tokenisation, and the normalised dataset in which all existing questions in the pre-processed text were normalised into short forms. Tables 2 and 3 summarise the performance of all algorithms on the pre-processed and normalised datasets, respectively.

**Table 2:** A Comparison of the classification performance of popular text classification algorithms and a basic CNN model on the pre-processed training set. The Best Score of Each Category is Highlighted in Bold.

Category	Algorithms			
	C4.5	SVM	NBM	CNN
ABSENT	0.967	**0.672**	0.967	1.066
MILD	0.428	0.337	**0.175**	0.446
MODERATE	0.709	0.836	0.773	**0.509**
SEVERE	0.885	1.052	**0.802**	0.916
Overall	0.747	0.724	**0.679**	0.734

**Table 3:** A comparison of the classification performance of popular text classification algorithms and a basic CNN model on the normalised training set. The Best Score for Each Category is Highlighted in Bold.

Category	Algorithms			
	C4.5	SVM	NBM	CNN
ABSENT	0.803	**0.656**	0.934	1.016
MILD	0.428	0.331	**0.229**	0.440
MODERATE	0.736	0.764	0.736	**0.509**
SEVERE	0.896	0.823	**0.740**	0.813
Overall	0.716	**0.643**	0.660	0.694

All methods performed better using the normalised dataset. For both datasets, NBM outperformed the others for the MILD and SEVERE classes, while SVM handles the ABSENT class rather well and acquires the best overall score on the normalised dataset. The results demonstrate that SVM can handle the imbalance issue shown in Table 1 better than others. On the other hand, the basic CNN model ranked third in both datasets, and performs better than the other algorithms in identifying the MODERATE class.

### 3.2 Effect of different input representations

**Fig 4:**
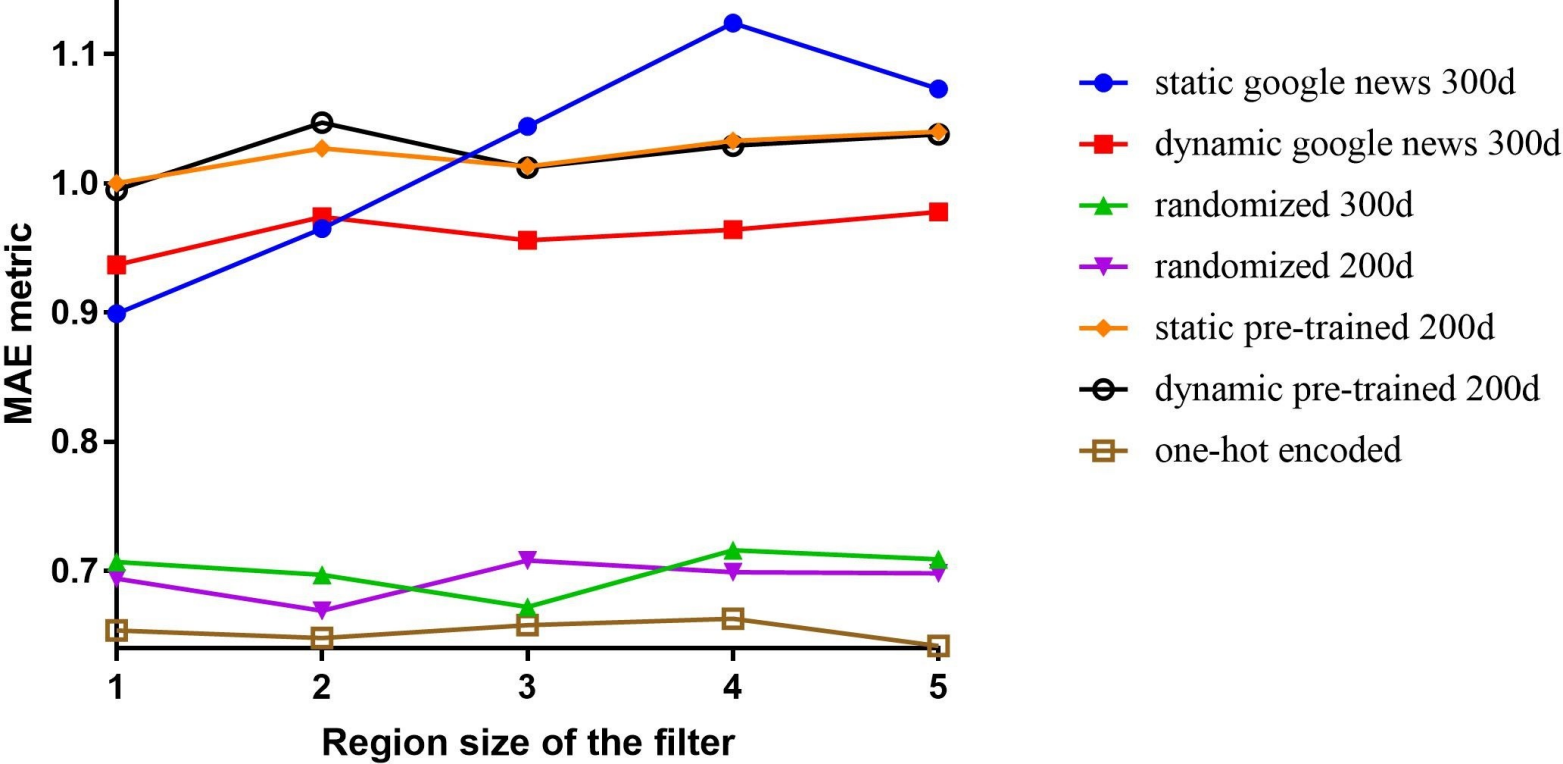
Effect of different input representations with different filter region sizes.

As described in the Methods section, the model illustrated in Fig 1 has the flexibility to swap the distributed representations of input words with different word representation vectors, including pre-trained word vectors. We replaced the randomly initialised word vectors used in the baseline configuration with different vectors such as one-hot encoded vectors, 300-dimensional randomly initialised vectors from Glorot uniform distribution [28], 200-dimensional pre-trained vectors from the entire CEGS N-GRID 2016 dataset (the full dataset contains 1,000 psychiatric evaluation records, including labelled and unlabelled records), and publicly available 300-dimensional embedding vectors (with a vocabulary size of three million words) induced from 100 billion words from Google News (GN). Both the GN and our pre-trained vectors were trained using word2vec [29]. For words absent in the pre-trained vectors, vectors with randomly initialised values were assigned. For models with pre-trained embedding layers, we applied two configurations: a static configuration in which all words, including unknown ones, were kept static, and only the other parameters of the model were learned; and a dynamic configuration in which the pre-trained vectors were fine-tuned with backpropagation. In addition, we varied the region size from 1 to 5. A comparison of the overall MAE according to the vectors and region sizes on the normalized training set is depicted in Fig 4.

The results show that configurations with fine-tuned pre-trained vectors have stable MAEs, which are generally better than those of static configurations. Previous research has revealed that the corpus domain for generating the word embedding is more important than the size of the training corpus [30]. Nevertheless, experiment results indicated that the models with GN-based embedding layers performed better than those with embedding layers trained from the CEGS N-GRID 2016 dataset, perhaps because the CEGS N-GRID 2016 dataset is too small to infer reliable representations of words.

Regarding the dimensionality of the embedding vectors, the model with the randomly initialised 200-dimensional vectors outperformed the one with 300-dimensional vectors. Furthermore, we noticed that the configurations with pre-trained vectors did not outperform those with randomly initialised vectors. In fact, the configuration with one-hot fixed vectors achieved the best overall MAE for all studied region sizes. Thus, we focus on models with one-hot-fixed embedding layers in the following experiments.

### 3.3 Effects of the numbers of feature maps and parallel CNN architectures

**Fig 5:**
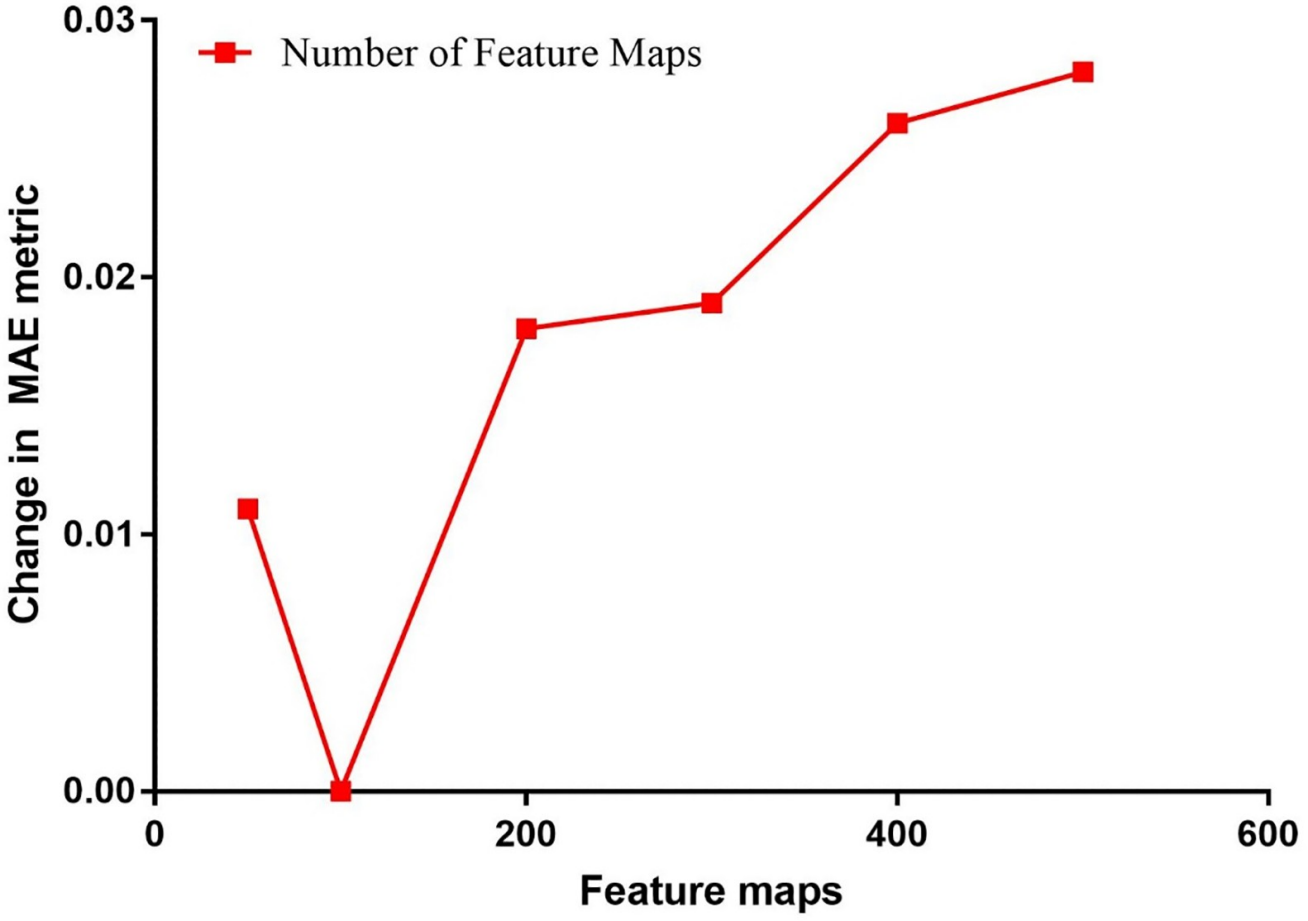
Effect of the number of feature maps measured using change in MAE.

We first explored the effects of the number of feature maps using CV in Fig 5. The model with a region size of 2 was selected as the baseline model, which achieved a MAE of 0.648 on the training set using CV. The number of feature maps was changed from 50 to 500 for each region size relative to the baseline model to understand the impact. All other parameters were held constant.

To study the influence of parallel CNN architectures, we combined the baseline model with different filter region sizes in the convolution layers, and fixed the number of feature maps for each region size at 100 (Table 4). Since we are only interested in the overall trend for change in MAE, instead of the absolute performance of each architecture, both Fig 5 and Table 4 displays the change in MAE in comparison to the baseline model. In Table 4, we use the notation {*n*} to indicate the architecture of the parallel CNN. For example, {2} is the baseline model. {2, 5} is a parallel CNN composing of two filters with region sizes 2 and 5. {1, 2, 5} is a parallel CNN composing of three filters with region sizes 1, 2 and 5.

**Table 4:** Effect of parallel CNN models measured using change in MAE.

Parallel CNN Configuration (filter regions)	Change in MAE metric
{2,2}	0.024
{2,5}	0.003
{5,5}	0.012
{1,2,5}	-0.004
{2,2,2}	0.002
{5,5,5}	0.017

We can observed from the above results that the optimal number of feature maps for the baseline CNN model with a region size of two was 100. Increasing the number of maps beyond 100 decreased performance and lengthy training times. This was consistently observed even after employing the dropout technique to avoid overfitting on the training set [31]. For parallel architectures, the MAE of the baseline model was reduced by 0.004 when combined with region 1 and 5. The best parallel architecture model ({1, 2, 5}) with an MAE of 0.644 outperformed that of NBM (0.660) and C4.5 (0.716).

### 3.4 Performance comparison of different region sizes and feature map numbers

**Fig 6:**
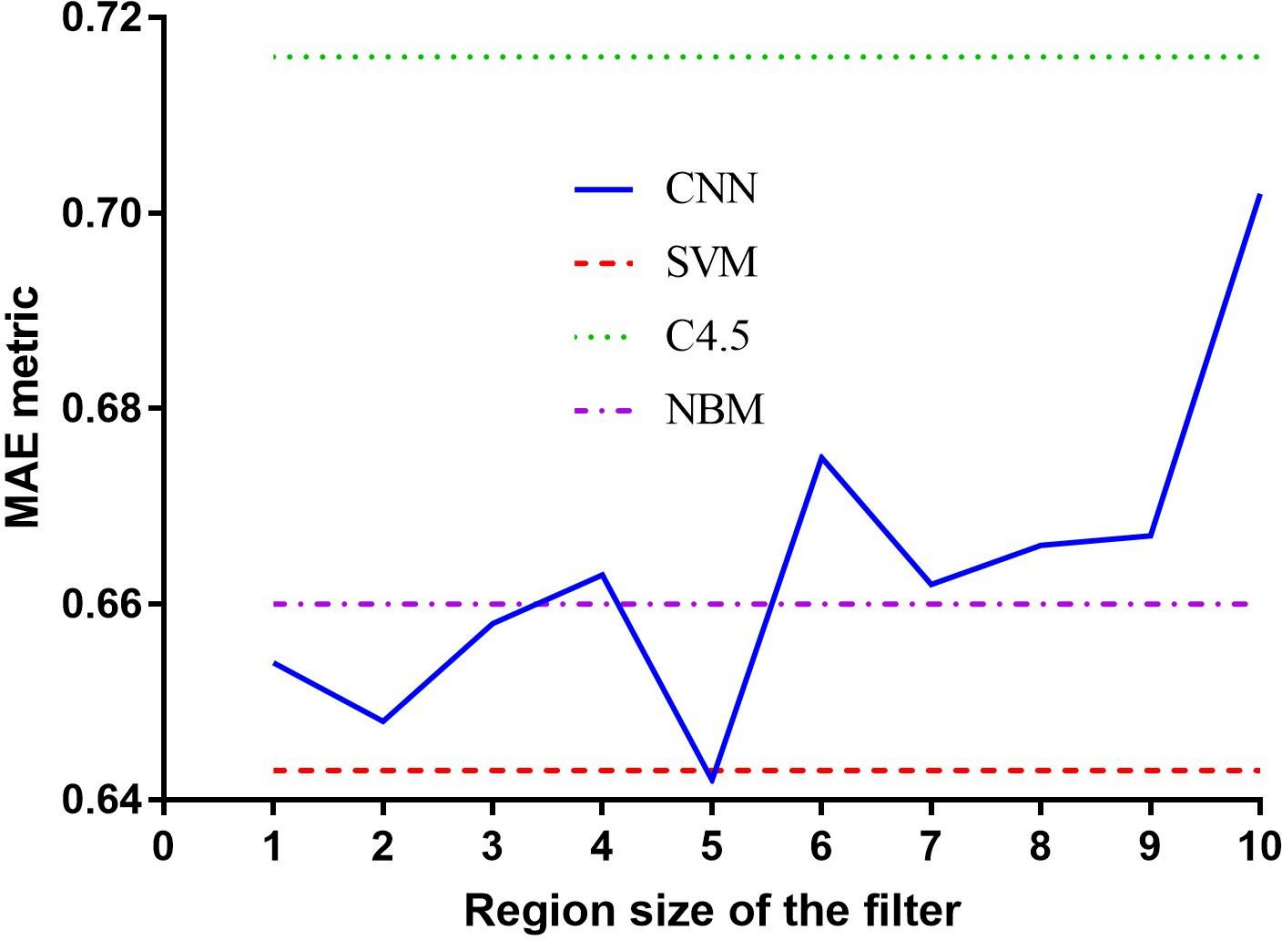
Performance comparison of CNN models with different region sizes and other baseline models.

We extended the model used in the previous section to investigate the impact of different region sizes. The model used 100 feature maps with a learning rate of 0.001. We performed a coarse grid search of region sizes ranging from 1 to 10 relative to the baseline individually, with other parameters held as constants. CV Results on the normalised training set are shown in Fig 6, suggesting that a reasonable range for the task may be from 1 to 5. The optimal single region size for the CNN model was 5, with which the model achieved an overall MAE of 0.642 and outperformed the best baseline model (the SVM).

### 3.5 Effect of chunk-max pooling CNNs

Further experiments were performed to examine the effect of a chunk-max pooling architecture. A model with a filter region size of 5 and 100 feature maps was selected as the baseline, as it had performed best in the previous experiment. We replaced the max pooling layer of the baseline model with chunk-max pooling layers, and the learning rate for all chunk-max pooling CNNs was set to 0.1. We inspected chunk sizes of 10, 20, 30, 40, 50, 75, 100, 150, 200, and 300, and held all other configurations constant.

**Fig 7:**
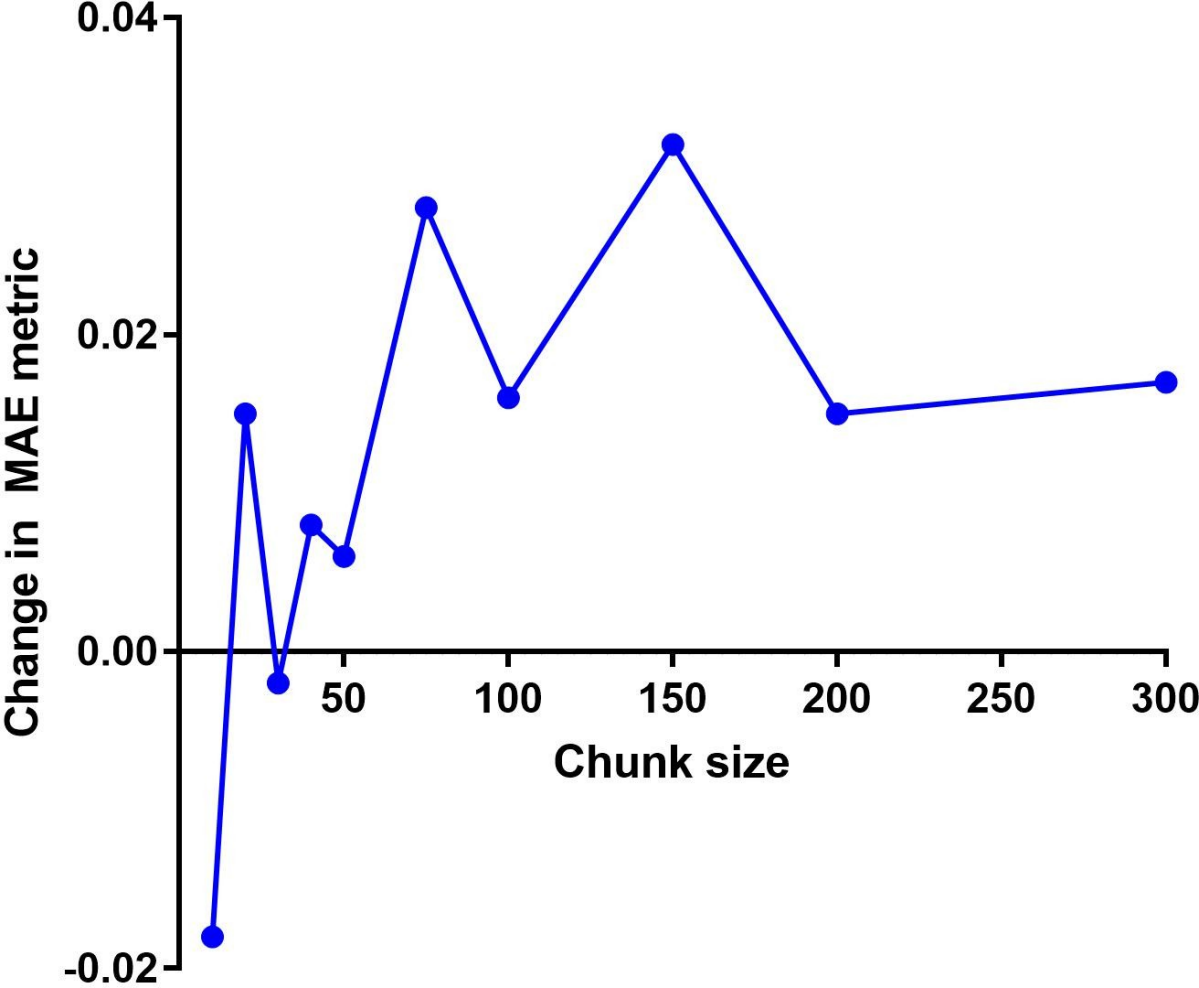
Changes in MAEs for different chunk-max pooling architectures.

In Fig 7, the chunk-max pooling architectures outperformed the baseline model when the chunk size was small. The best MAE (0.624) was achieved by the model with a chunk size of 10. This improvement may result from the ability of the architecture to retain the order in which features occur.

### 3.6 Performance on the test set

Finally, we present the performance of the proposed CNN models and the other three text classification algorithms on the CEGS N-GRID 2016 test dataset in Table 5. The two CNN models with the best performance were selected for this comparison: the max-pooling CNN model with a region size of 5 and 100 feature maps (denoted as m-CNN) and the chunk-max pooling CNN model with a chunk size of 10 (denoted as c-m CNN). The NBM and SVM performed best on the MILD and ABSENT classes, respectively. However, the two CNN models performed better in the SEVERE class and achieved greater overall MAEs on the test set.

**Table 5:** Results on the CEGS N-GRID 2016 test set. The CNN Models with Dropout are Denoted with an Apostrophe.

Classification	C4.5	SVM	NBM	m-CNN	c-m CNN	m-CNN’	c-m CNN’
ABSENT	0.968	**0.645**	0.645	0.871	0.742	0.677	0.677
MILD	0.430	0.360	**0.244**	0.384	0.407	0.337	0.419
MODERATE	0.630	0.761	0.652	**0.413**	0.522	0.5	0.478
SEVERE	0.925	0.698	0.792	0.642	0.660	0.642	**0.623**
Overall	0.738	0.616	0.583	0.577	0.583	**0.539**	0.549

Table 5 also includes the performance of the two CNN models (denoted as m-CNN’ and c-m CNN’, respectively) after applying a dropout rate of 0.1 to the output of the max-pool layers as a means of regularisation. Both the m-CNN and c-m CNN benefitted from utilizing the dropout, and m-CNN’ achieved the best MAE, of 0.539. A possible explanation for this improvement is that the dropout technique can help our one-hot encoding to avoid overfitting.

## 4 Discussion

### 4.1 Comparison of the predicted severity levels of the CNN models and the baseline models

From Eq 7, we know that MAE measures the average magnitude of the predicted errors among the four classes considered in the study (absent, mild, moderate and severe). Therefore, in our evaluation a MAE of one point indicates that the classifier’s prediction is different from the gold annotation with one scale. For instance, if the gold annotation for a record is moderate, the classifier with MAE of one may have predicted the record as either mild or severe. From the perspective of psychiatrists, in order to guarantee patient and personal safety, misclassification of a severe case as mild or even absent is not allowed since it may lead to a delay in hospital admissions. By contrast, classifying absent cases as moderate ones is somewhat acceptable.

From the results of MAE on both the CV of the training set and the test set, we have observed that the CNN-based models generally had a better performance, in particular on the categories of the moderate and severe classes as indicated by the lower MAE values. Although decision tree models like C4.5 used in this study can yield a better outcome representation for human understanding, their poor discriminability for the absent and severe classes explains why the CNN models should be considered as an alternative even if they were criticized for their difficult interpretation.

**Fig 8:**
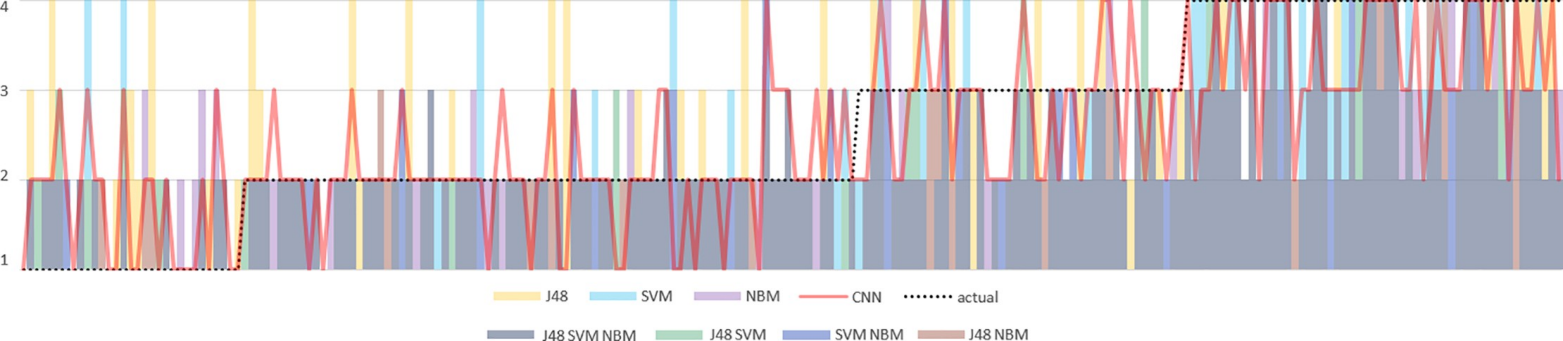
Bar-line combination chart of the gold scores (actual) and the predicted scores of each record on the test set. The horizontal axis is the records sorted according to their scores in an ascending order, and the vertical axis is the assigned scores ranging from 1 to 4.

Inspired by the error analysis conducted by Duda, Kosmicki [32] and Moreau and Vogel [33], we compared the predicted scores with the gold scores assigned by psychiatric experts by combing the assigned scores for each record in Fig 8. We sorted the records in the test set according to their assigned gold scores in an ascending order, which were depicted as the dotted line in Fig 8. In the combined bar and line chart, the scores predicted by baseline algorithms were highlighted with different colours in the bar chart, and the predictions of the best CNN model shown in Table 5 were displayed as the red solid line. This figure enables us to understand the false positive/negative rates of the studied algorithms and the distance scale for each article.

For records assigned with the absent score, we can observe from the colourful bars in Fig 8 that SVM has the least average score distance while C4.5 has the largest distance. We also noticed that both the NBM and CNN models never misclassify the absent records as the severe class. For the mild class, NBM apparently outperforms the others since only a few records are associated with the bar in purple. Additionally, we found that some records were misclassified as moderate or severe classes by all of the models. As for the moderate and severe classes, the CNN model had less score ranges. When a wrong prediction was made, it tends to assign the adjacent class with the nearest distance (i.e., mild or severe for moderate, and moderate for severe). Moreover, the moderate and severe classes were never misclassified as absent by the CNN model. As a whole, we believe that the CNN model should be preferred over the others for classification in terms of patient and personal safety.

### 4.2 Error analysis

#### 4.2.1 Analysis of the models’ outputs

As demonstrated in Fig 8, there are some records in each class that cannot be correctly classified by all of the developed models. The mild class constituted 9.3% of the misclassified records, which is the least among all classes probably because it is the class with the most training cases. Nevertheless, we still noticed a significant peak in the region of the mild class in Fig 8. We examined the record and found that the patient expressed his concern over the bipolar disorder of his son (refer to the example of the MILD class in Table 6.), and also mentioned usage of multiple addictive substances such as marijuana. However, the clinician diagnosis did not recommend further substance abuse treatment. This example demonstrates that the medical history information may not be helpful without describing the frequency of substance usage and whether the mentioned event is the main focus of a treatment. Unfortunately, the competence of all developed models is affected by this issue which may be owing to the insufficient training examples.

**Table 6:** Examples of vague descriptions across different classes.

Class	Example Context
ABSENT	The patient has a history of depression and anxiety that started approx 20 years ago, and has been effectively controlled with psychiatric medication. The patient reports that he started to see therapist for his depression at age 16 when he tried to commit suicide. … His interest include a weekly game night with friends that he attends, even when depressed.
MILD	Has also noted that his own mood has been low in periods where he is more worried about his son (described him as manic-depressive, "lives like a pig," …
MODERATE	Pt currently meets criteria for PTSD and likely TBI. He reports several re-experience, avoidance, and hyperarousal symptoms that cause significant distress and impairment (see below). In addition he has experienced numerous head injuries that likely contribute to his reports of poor concentration, low distress tolerance, headaches, and memory difficulties. Pt reports recen history of etoh abuse … The pt is interested in indivdiual therapy and/or couples therapy focused on parenting conerns.
SEVERE	He has no prior periods of either depression or mania, but per family last year when he used cannabis for the first time, it made him feel really high for several days…

The absent and moderate classes were much more difficult to identify, as 32% and 36.9% of the absent and moderate examples were misclassified, respectively. After scrutinizing these records, we discovered that 91% of the patients in the absent records presented high severity for symptoms or disorders related to anxiety or depression, which belong to the negative valence domain. The subtle distinction between the positive and negative valence domains make their classification extremely challenging. For example, a depressed patient should be scored absent, but a patient who is depressed with a loss of interest in activities that needs to be treated should be scores at least moderate (*e*.*g*. people who need an intervention to get out of bed). At times, records may contain references to previous events which increase the vagueness of the decision. In addition, discriminating between the moderate and severe classes is also difficult as it requires the recognition of several contributing factors such as the patient’s history of illness, social history, habits…etc. Some records of patients with extremely complex conditions document various relevant medical information like syndromes reflected in multiple domains result in noisy information (refer to the example in Table 6) and require extensive understanding of the content for interpretation. To conduct such profound understanding of texts, different representation techniques to capture natural language syntax and semantics plays an important role in this task [34, 35]. Finally, we noticed that some records contain only question-answer pairs without additional narratives, which also hinder the analysis of their content.

For records in the severe class, 18.9% of the records were misclassified by all models. Some of these records contain very limited positive valence signals with many negative valence signals, and the characteristics of these records may confuse the developed models in either the training or the predicting phase. Furthermore, in some records patients may refer to another person’s positive symptoms, describe previous positive symptoms, or express several negations regarding symptoms and substances. Content as such can also mislead the models’ judgements. Incorporation of section recognition techniques [36] and negation detection methods [37, 38] is likely to lead to an improvement in system performance. Table 6 lists some example descriptions related to the issues from different classes that were studied.

#### 4.2.2 Analysis of the effectiveness of word representations

In Fig 4, we observed that, for severity classification of positive valence symptoms in psychiatric evaluation records, a CNN model with one-hot encoding vectors obtained the best overall MAE compared to other word representation methods. To better comprehend the rationale underlying this result, we conducted two additional experiments.

**Fig 9:**
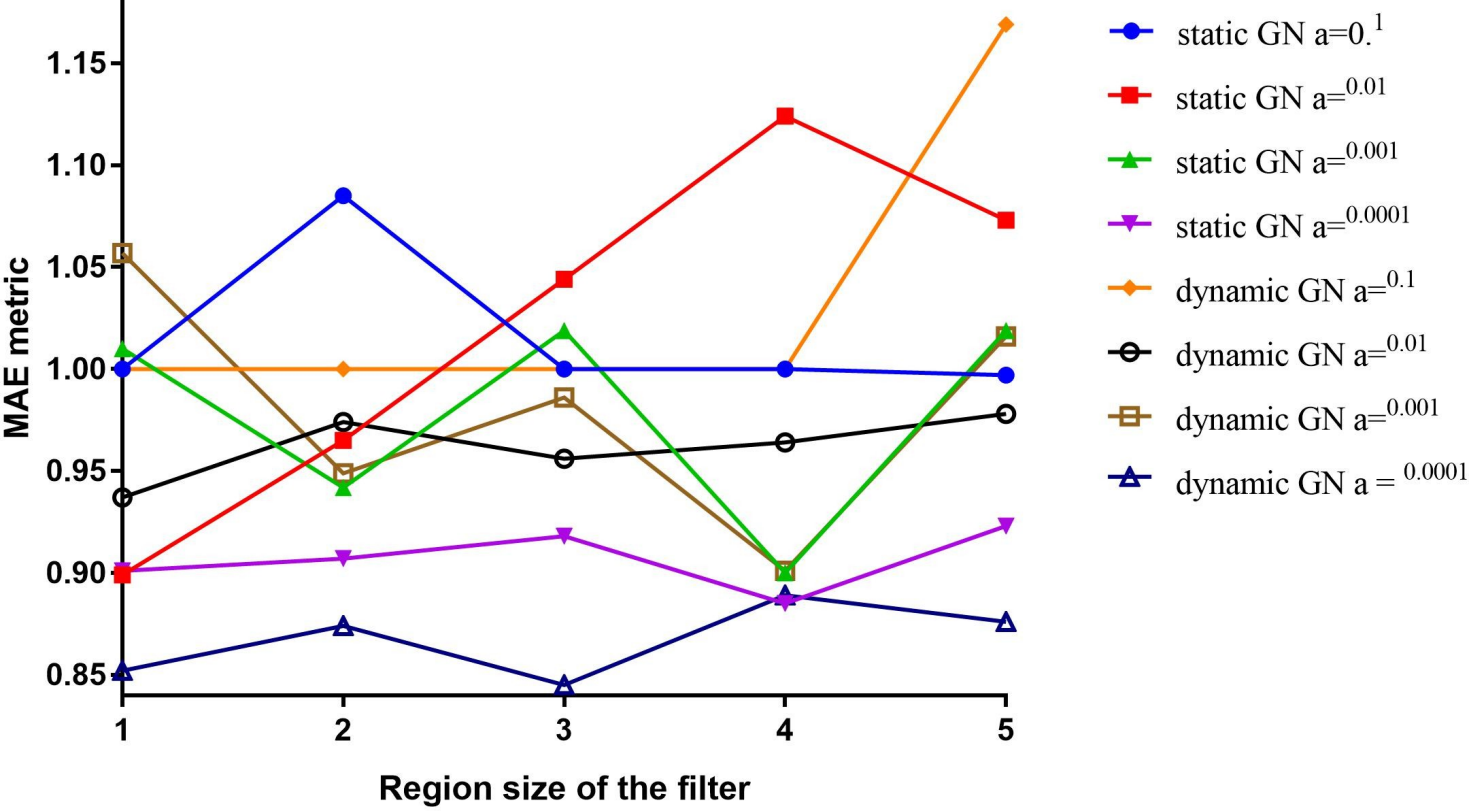
Effects of different learning rates and region sizes on the GN-based models.

First, we noted that GN models did not perform well on the training set, which we presumed was due to the learning rate α we selected. The best learning rate for a model may depend on several factors, such as the architecture and purpose of the model being optimised. A larger value of α could help a model locate a global optimum, whereas a smaller value of α may guide the model to a local optimum. To retrieve the optimal α for our GN models, we progressively reduced the learning rate from 0.1 to 0.0001, the results of which are displayed in Fig 9. Of the learning rates employed, both static and dynamic GN configurations with the lowest value of α (0.0001) achieved the best MAEs.

Subsequently, we examined the word representation used in our models. One of the main advantages of word embedding is that after representing words as vectors, similar words tend to have similar vectors, so that the similarity between words could correlate with the cosine similarity between the vectors of these words [39]. As shown in Tables 2 and 3, all algorithms performed better on the normalised dataset. Question normalisation seems to be an essential step, since questions in initial psychiatric evaluations tends to be long.

**Fig 10:**
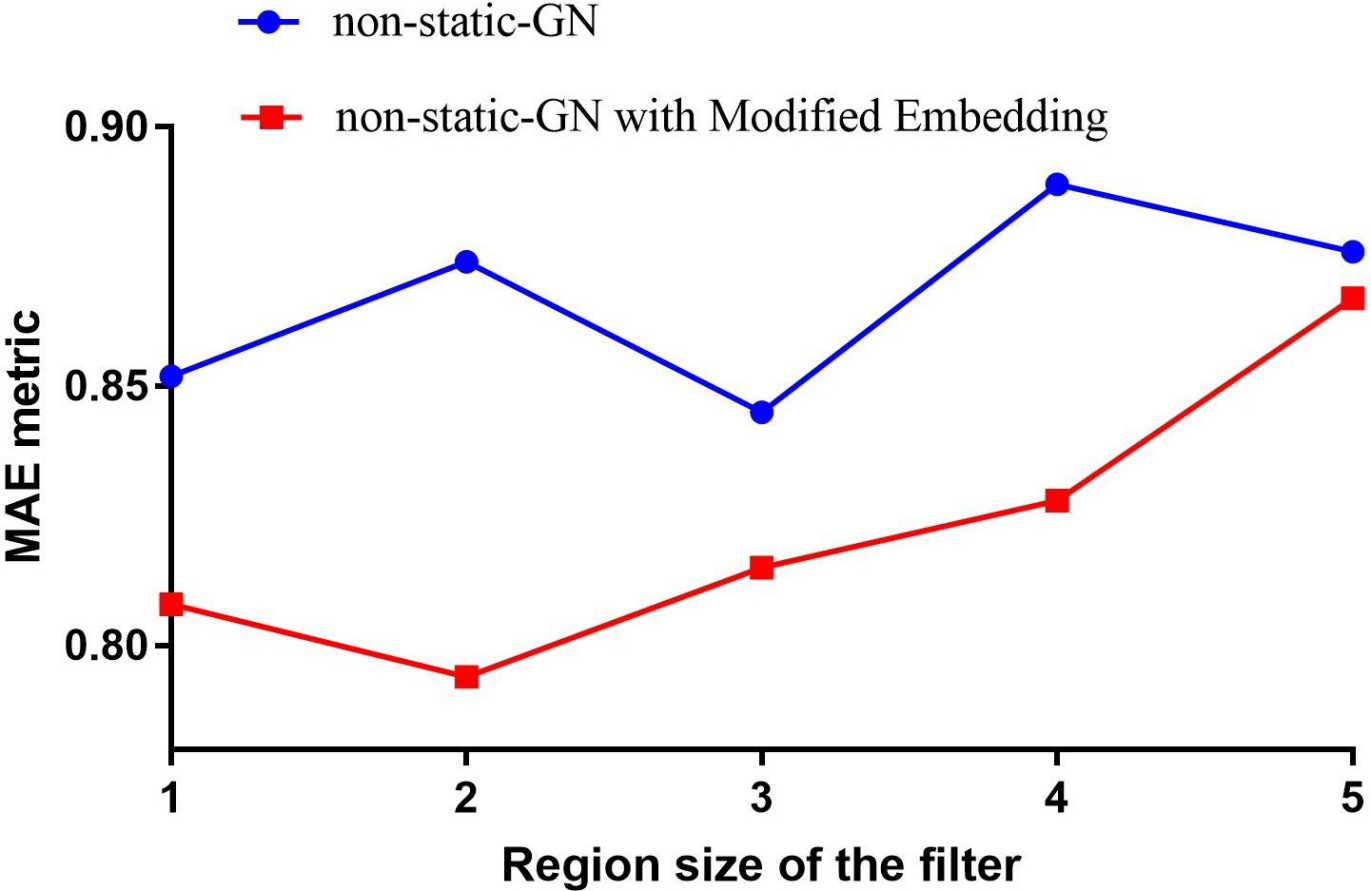
Performance comparison of dynamic GN with different embedding representations.

We extracted the words used for questions and responses and checked their similarity after representing them as vectors with GN and our pre-trained embedding. We found that the word ‘Yes’ was listed as the twelfth-most similar word to ‘No’, with a cosine distance of 0.491. This similarity is even higher than that between the words ‘none’ and ‘No’ in GN embedding. We further observed that when looking for words similar ‘Q2’, GN returned ‘Q3’ (0.973), ‘Q1’ (0.965), and ‘Q4’ (0.926) as the top three words. As described in the pre-processing section, we normalised questions into these short forms, which seems to have resulted in several questions being mapped to nearby points. We believe that this issue led to inefficiency in applying pre-trained word vectors in our task. Consequently, we conducted an additional experiment to explore possible improvements in performance after breaking the similarity among these words. A list of words was created to include words that were frequently used in all questions, as well as responses such as ‘Yes’, ‘No’, ‘None’, and ‘Uncertain’. The listed words were considered to be out-of-vocabulary words, and were assigned with randomly initialised vectors instead of using the corresponding pre-trained vectors. The results of the experiment are displayed in Fig 10.

As indicated in Fig 10, the performance of dynamic GN models could be improved with different word representations. The results concur with the point given by Severyn and Moschitti [40] in which they stated that the initialization of model parameters is very important for training an accurate CNN model. Feeding CNN with a high quality embedding is critical because it makes the training starting from a good point. However, hyper-parameter optimisation is a main research problem when using CNNs, since there are no definite, explicit ways to select optimal parameters. For example, the current activation function used in our model is ReLU, but there are several alternatives, such as leaky ReLU [41], parametric ReLU [42], maxout [43], and tanh. We will consider exploiting these alternatives in future work.

### 4.3 Comparison with the CEGS N-GRID shared task results

The CEGS N-GRID shared task in 2016 had 24 teams submitting 65 runs. In addition to the regular MAEs, the CEGS N-GRID shared task proposed to use a new normalized macro-average MAE (NMAE) metric to report the overall performance across the four classes [18]. NMAE is a customized MAE by normalizing the deviations, and macro-averaging the errors across the classes according to Eq 8

$$\mathrm{NMAE}=1-\frac{1}{\left|C\right|}\sum_{j=1}^{\left|C\right|}\frac{1}{\left|D_{j}\right|}\sum_{x_{i}\in D_{j}}\frac{\left|p\left(x_{i}\right)-y_{i}\right|}{\max\left(y_{i}-1,\left|C\right|-y_{i}\right)}$$ (8)

In the CEGS N-GRID shared task, the average NMAE among all the submissions was 0.771 with a median of 0.776. The best performing run scored 0.863, whereas the lowest one scored 0.525. Our two best performed CNN architectures illustrated in Table 5 achieve NMAE scores of 0.780 and 0.785, respectively.

All the top performed teams in the shared task used ensemble strategies along with feature engineering to boost the performance of their systems. For example, Kagan, Subrahmanian [14] employed association rule mining method to develop binary features and create 22 machine learning-based classifiers for ensemble learning. Their system achieved the best NMAE of 0.863 in the shared task. For each psychiatric evaluation record, Goodwin, Maldonado [44] extracted 568 features and proposed a hybrid model combining ridge regression and random forest models. The NMAE of the hybrid model is 0.841, which was ranked in the second. Rios and Kavuluru [17] proposed a new CNN architecture with an ordinal loss function and created an ensemble of that and linear models including SVMs, logistic regression, ridge regression, and logistic ordinal regression. Furthermore, they encoded auxiliary features such as histories of suicidal/violent behaviour, and prior inpatient/outpatient treatments, in the max-pooled vectors to incorporate the psychopathology related information in their CNN model. Their approach was ranked third in the shared task with an NMAE of 0.839. Hsieh, Chang [16] also employed an ensemble of NBM, CNN and log-likelihood ratio-based logistic regression classifier. Their system achieved an NMAE of 0.757. Clark, Wellner [45] formulated the task as a text classification problem and employed a neural network architecture based on multi-layered perceptron with selected features such as unigrams, DSM codes and their frequency, and the frequency of “Yes” responses in questions. The NMAE of their best model is 0.779.

Compared to other similar studies, the key contribution of our study is that we investigated several classic CNN architectures and their variances and explored various possible configurations. Exploring and tuning the possible configurations to optimize the performance is an extremely expensive task. We empirically identified the settings for hyper-parameters/architectures and possible issues, such as the use of word embedding, which should be considered while adapting classical CNN architectures for similar document classification task. Furthermore, unlike the top-ranked teams in the CEGS N-GRID shared task which made great attempt to separate semi-structured data (e.g. question-answer pairs) from unstructured text, we studied the ability of the CNN-based models in ingesting the entire psychiatric records for severity classification, which made our results reflect the true discriminability if we applied such models to records with different semi-structured format.

## 5 Conclusion

This work studied the document classification problem of positive valence symptom severity in initial psychiatric evaluation records containing not only narrative descriptions, but also semi-structured text, such as attribute–value or question–answer pairs. We demonstrated that normalising the semi-structured component of the records can improve the MAE of each classification algorithm we examined. Without advanced feature engineering or feature selection, our results indicate that CNN-based approaches provide a comparable solution for classifying positive valence symptom severity in initial psychiatric evaluation records. Although word embedding is well known for its ability to capture similarity between words at relatively low dimensions, directly including a pre-trained embedding layer in our CNN model did not improve classification performance. This may result from the inability of our word embedding to capture antonyms leading to a low quality representation. Our results suggest that if one would like to apply CNN-based models in classifying data involved question-answer pairs without sophisticated pre-processing, one-hot encoded vectors or advanced word embedding technologies other than word2vec are a better choice for representing words.

In a nutshell, despite the advantages of employing CNN models, the challenge of parameter optimisation and obtaining high quality embeddings remains. For instance, slight changes in network architecture may require different hyper-parameters, which makes the models hard to tune. The selection of learning rates and activation functions, and the problem of overfitting, are also critical factors that affect the performance of the models we developed. The results of our error analysis also demonstrate the importance for more profound understanding of the content which may require some expert knowledge in the domain. We would like to address these research questions in the future and explore better representation technologies to capture the syntax and semantics of the content in a more appropriate manner.

## Supporting information

S1 Fig Age distribution of the CEGS N-GRID 2016 dataset. (TIF)

S2 Fig Gender distribution of the CEGS N-GRID 2016 dataset. (TIF)

S1 File Appendix A. (PDF)

S2 File Appendix B. (PDF)
