# Supplementary figures and images for: Assessing the severity of positive valence symptoms in initial psychiatric evaluation records: Should we use convolutional neural networks?
Source: PLoS One. 2018 Oct 16;13(10):e0204493. doi: 10.1371/journal.pone.0204493 (PMC6191093; doi:10.1371/journal.pone.0204493)

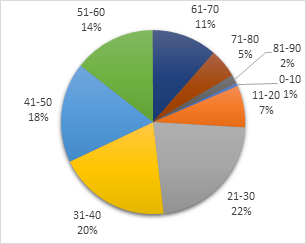

Supplement: S1 Fig — (TIF) [file pone.0204493.s001.tif]

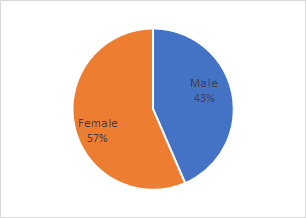

Supplement: S2 Fig — (TIF) [file pone.0204493.s002.tif]
