# Supplementary material for: Assessing the severity of positive valence symptoms in initial psychiatric evaluation records: Should we use convolutional neural networks?
Source: PLoS One. 2018 Oct 16;13(10):e0204493. doi: 10.1371/journal.pone.0204493 (PMC6191093; doi:10.1371/journal.pone.0204493)
Supplement: S1 File — (PDF) [file pone.0204493.s003.pdf]

Appendix A

For features used by baseline algorithms include C4.5, support vector machine (SVM), and naïve Bayes multinomial (NBM), we evaluated their effectiveness by measuring their information gain with respect to the four classes by Eq. (A.1) and filtered out lower-ranking (less useful) features.

$$\mathrm{InfoGain}(Class, Attribute) = \mathrm{H}(Class) - \mathrm{H}(Class|Attribute) \quad \text{(A.1)}$$

In Eq. (A.1), H specifies the entropy, which can be written as Eq. (A.2).

$$\mathrm{H}(P) = \mathrm{H}(P_1, P_2, \ldots, P_n) = -\sum_{i=1}^{n} P_i \log P_i \quad \text{(A.2)}$$
