# Supplementary material for: Assessing the severity of positive valence symptoms in initial psychiatric evaluation records: Should we use convolutional neural networks?
Source: PLoS One. 2018 Oct 16;13(10):e0204493. doi: 10.1371/journal.pone.0204493 (PMC6191093; doi:10.1371/journal.pone.0204493)
Supplement: S2 File — (PDF) [file pone.0204493.s004.pdf]

Appendix B

The demographic information of the used datasets including the distributions of gender and age is shown in Figure 1 and 2, respectively. Note that the information was automatically extracted by our text mining pipeline (Dai et al., 2017) without manual validation, which was developed for mining factors strongly associated with violent behaviours among psychiatric patients.

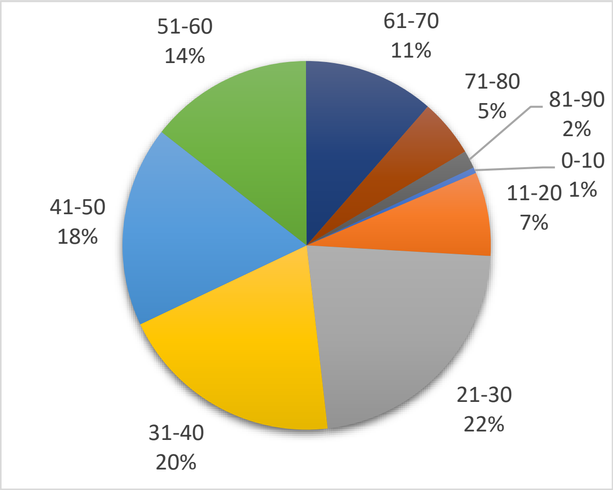

Figure 1. Age distribution of the CEGS N-GRID 2016 dataset.

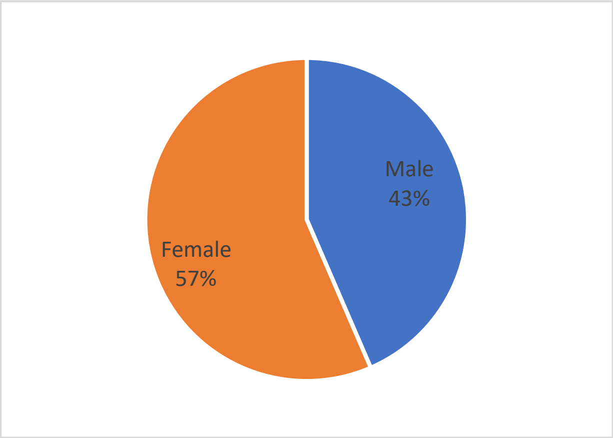

Figure 2. Gender distribution of the CEGS N-GRID 2016 dataset.
